# The evaluation of a rectal cancer decision aid and the factors influencing its implementation in clinical practice

**DOI:** 10.1186/1471-2482-14-16

**Published:** 2014-03-21

**Authors:** Robert Wu, Robin Boushey, Beth Potter, Dawn Stacey

**Affiliations:** 1 The Ottawa Hospital General Campus, 501 Smyth Rd., Ottawa, Ontario K1H 8L6, Canada; 2 Department of Surgery, The Ottawa Hospital General Campus, 501 Smyth Rd., Rm 1617, Critical Care Wing, Ottawa, ON K1H8L6, Canada; 3 Department of Epidemiology & Community Medicine, The University of Ottawa, 451 Smyth Rd. RGN 3230F, Ottawa, ON K1H8M5, Canada; 4 Department of Epidemiology & Community Medicine, Centre for Practice Changing Research, Ottawa Hospital Research Institute, The Ottawa Hospital General Campus, 501 Smyth Rd., Ottawa, ON K1H8L6, Canada

**Keywords:** Rectal cancer, Surgery, Patient centered care, Decision aid, Shared decision making

## Abstract

**Background:**

Colorectal cancer is common in North America. Two surgical options exist for rectal cancer patients: low anterior resection with re-establishment of bowel continuity, and abdominoperineal resection with a permanent stoma. A rectal cancer decision aid was developed using the International Patient Decision Aid Standards to facilitate patients being more actively involved in making this decision with the surgeon. The overall aim of this study is to evaluate this decision aid and explore barriers and facilitators to implementing in clinical practice.

**Methods:**

First, a pre- and post- study will be guided by the Ottawa Decision Support Framework. Eligible patients from a colorectal cancer center include: 1) adult patients diagnosed with rectal cancer, 2) tumour at a maximum of 10 cm from anal verge, and 3) surgeon screened candidates eligible to consider both low anterior resection and abdominoperineal resection. Patients will be given a paper-version and online link to the decision aid to review at home. Using validated tools, the primary outcomes will be decisional conflict and knowledge of surgical options. Secondary outcomes will be patient’s preference, values associated with options, readiness for decision-making, acceptability of the decision aid, and feasibility of its implementation in clinical practice. Proposed analysis includes paired t-test, Wilcoxon, and descriptive statistics.

Second, a survey will be conducted to identify the barriers and facilitators of using the decision aid in clinical practice. Eligible participants include Canadian surgeons working with rectal cancer patients. Surgeons will be given a pre-notification, questionnaire, and three reminders. The survey package will include the patient decision aid and a facilitators and barriers survey previously validated among physicians and nurses. Principal component analysis will be performed to determine common themes, and logistic regression will be used to identify variables associated with the intention to use the decision aid.

**Discussion:**

This study will evaluate the impact of the rectal cancer decision aid on patients and help with planning strategies to overcome barriers and facilitate implementation of the decision aid in routine clinical practice. To our knowledge this is the first study designed to evaluate a decision aid in the field of colorectal surgery.

## Background

Colorectal cancer is the third most common cancer accounting for 13% all new cancer diagnoses in Canada [1]. Rectal cancer comprises any tumor in the last 15 cm of the large intestine. The treatment of rectal cancer is especially challenging given the pelvic anatomy and the related muscles and nerves involved in sphincter control, as well as bladder and sexual functions. Two surgical therapies exist for rectal cancer patients: low anterior resection (LAR) with re-establishment of bowel continuity, and abdominoperineal resection (APR) with a permanent stoma. Each procedure presents a particular set of benefits and risks. While LAR is associated with higher risks of leakage at the intestinal re-connection and fecal incontinence; APR is associated with higher risks of stoma related hernia, prolapse, skin damage, and surgery related infections [2]. LAR combined with neoadjuvant therapy has seen a significant rise in surgical practices, especially given the non-inferior oncological outcomes in patient survival when compared to APR [2]. Although it was once believed that avoiding a permanent stoma would improve quality of life, recent literature has questioned the validity of that claim [3-6].

A meta-analysis identified APR as having better global psychological and emotional scores, while LAR was associated with better physical symptom and pain scores. However, results for overall quality of life (QoL), despite being measured by various validated instruments, were consistently equivocal [7]. Similarly, a recently updated Cochrane Review has revealed that low anterior resection (LAR) did not lead to superior QoL [8]. Given the equivalent survival outcomes, and the need to weigh QoL outcomes, the decision for rectal cancer surgery is therefore a value-laden one that deserves the consideration of the patient perspective.

Evidence suggests that patients have played a passive role in the decision process: the decision traditionally relies on three factors including 1) patient’s baseline functional status, 2) surgeon preference, and 3) tumour characteristics [7]. Greater involvement of patients in decision-making may lead to higher quality decisions congruent with patient preferences.

Shared decision making (SDM) is a model that seeks to include both the patients and their healthcare providers in the decision making process [9]. It encourages patients to play an active role in decisions concerning their health, which is a goal of patient-centered care [10]. This model of care has been endorsed by the recent Salzburg Declaration [11] and the US National Academy recommendations on State and Federal SDM implementation [12]. SDM can be facilitated by patient decision aids (PtDA), which are defined as interventions designed to help people make specific and deliberative choices among options by providing information on the options and outcomes relevant to the patient’s health status [13]. The effectiveness of PtDA has been demonstrated in at least three separate systematic reviews [14,15]. They have been shown to improve patient knowledge, lower decisional conflict related to feeling uninformed and unclear about personal values, reduce the proportion of people who were passive in decision making post-intervention, and improve agreement between patient values and health care option chosen [14]. Furthermore, exposure to decision aids can lead to a higher proportion of people with accurate risk perceptions [14]. Despite the demonstrated value of decision aids, a thorough search through the Ottawa Decision Support A to Z Inventory and Decision Aid Library Inventory (DALI) [16], comprehensive catalogues of decision aids, did not return any decision tools to engage rectal cancer patients in the decision-making process regarding the most appropriate surgical treatment for them.

In 2003, consensus standards for PtDAs were established by the International Patient Decision Aid Standards Collaboration (IPDAS) [17]. Through the Delphi method, the IPDAS involved over 100 major stakeholders such as patients, practitioners, researchers and policy makers to develop the criteria for the assessment of the quality of PtDAs. The Ottawa Decision Support Framework (ODSF) is a decision making framework informed by cognitive, social, and organizational psychological theory that guides the assessment and development of PtDAs [18]. This framework contributed to the development of the IPDAS [19]. Guidelines [20] put forth by the ODSF have been used in the development of at least 31 decision aids that were involved in 24 randomized controlled trials [21]. Adhering to quality criteria set by the IPDAS and using the ODSF as a template, a rectal cancer PtDA was developed (Additional file 1) following three principal steps: 1) A systematic review to explore the long term side effects of rectal cancer surgeries; 2) Needs assessments on rectal cancer patients and colorectal surgeons; 3) Development of a decision aid. The systematic review identified that bowel dysfunction after surgical therapies was high [22]. The needs assessments identified a lack of awareness of surgical options and their outcomes, as well as a general lack of patient involvement in the decision making process [23]. The needs assessment also identified that despite the practitioners expressing commitment to shared decision making, their view of the population to which this could apply was limited. The need for a rectal cancer decision aid was identified to encourage quality communication between patients and their practitioners. The decision aid was then designed by integrating evidence-based knowledge on the benefits and risks associated with LAR and APR. Since development, its content and presentation have been reviewed by a patient and a surgeon, but it has not been formally evaluated.

As the rectal cancer decision aid has been developed, the current study is the next step with aims to evaluate and implement the decision aid. The first phase of the proposed study will continue to follow recommendations of the ODSF to evaluate the decision aid with patients at the point of decision making [20] via a pre- and post-DA design.

Adoption of evidence does not solely rely on the evaluation from patients, as decision making is a process that involves both the patients and the healthcare professionals. Patients’ input is necessary but their perception alone has not been sufficient to change clinical practice [24,25]. It is not surprising that even though patient decision aids may foster SDM [26,27], it has been shown that wide spread use in clinical practice has been poor [28-30]. Given the effectiveness of PtDAs, significantly more effort is needed to understand the factors preventing their integration into clinical practice [31]. The Knowledge-to-Action Cycle is a dynamic and reiterative guiding framework to outline strategies for implementation and application of knowledge [32,33]. It is a framework used by the Canadian Institutes of Health Research (CIHR) for transfer of research findings into practice [34]. It promotes the identification of potential barriers as a necessary step prior to developing an implementation strategy for the wide use of an intervention [32].

Furthermore to maintain practice change, there needs to be interventions designed to address the contextual factors that may affect the implementation [35]. A systematic review reported that the three most commonly cited barriers by health professionals are: 1) time pressure; 2) lack of SDM applicability due to patient characteristics; 3) lack of SDM applicability due to the clinical situation [24]. These results indicate that healthcare professionals in general are feeling mounting pressure from limited resources, and that practitioners may be selecting patients for SDM. This knowledge helps inform strategies for future implementation.

To address health professionals’ barriers to use the rectal cancer decision aid, the second phase of the proposed study will explore the issues perceived by the potential adopters—the surgeons working with rectal cancer patients in North America. The assessment of facilitators and barriers specific to the rectal cancer decision aid will help establish the factors associated with the healthcare professionals and the practice environment, and provide direction for selecting and tailoring implementation strategies.

Taken together, decision aids are a platform for shared decision making, an important goal for patient-centred care. Following the guidelines of the Ottawa Decision Support Framework, a rectal cancer surgery PtDA has been developed but not evaluated. In addition, the assessment on patients’ perspective alone is insufficient for the successful implementation of the decision aid in a clinical setting. Evidence translation into clinical practice needs to involve the healthcare professionals. The current study seeks to address these two issues by evaluating the decision aid on patients and exploring the barriers and facilitators of implementation.

### Study objectives

The current thesis study aims to 1) evaluate the effect of a decision aid on patients’ choice and decision-making process and 2) explore surgeons perception of the facilitators and barriers influencing implementation of the rectal cancer decision aid for patients who are considering low anterior resection versus abdominoperineal resection.

## Methods

### Part 1: pre- and post- PtDA study

Research questions:

1) What is the effect of a decision aid on patients’ choices and decision-making processes, among rectal cancer patients?

2) What is the acceptability of the rectal cancer decision aid among patients?

3) What is the feasibility of implementing the decision aid into usual clinical practice?

### Study design

A before and after study will be used to evaluate the decision aid. A review examining PtDA evaluative studies showed that before/after studies and randomized controlled trials have both demonstrated DA effect on choice, patient comfort with decision making, outcomes of decisions, and patient acceptability [36]. In addition, the rectal cancer PtDA was developed according to the ODSF, which has a strong theoretical foundation that has been extensively validated [19]. Given the Ottawa framework has already been evaluated over 20 RCT’s, a pre- and post- DA design was identified as appropriate to explore PtDA effect on decisional conflict before and after intervention, acceptability as perceived by patients, and the feasibility of implementing the DA into usual clinical practice.

### Participants

The study will take place in a tertiary care hospital serving a population of 1.3 million people. The Colorectal Assessment Centre at the Ottawa Hospital receives on average 12 rectal cancer patient referrals per month. Approximately half (6/12) of these patients would be eligible for the study. Eligible participants are 1) patients with newly diagnosed stage I-III rectal cancer confirmed by biopsy; 2) tumor located at a maximum of 10 cm proximal to anal verge; 3) surgeon screened candidates eligible to consider LAR and APR; 4) age ≥ 18; and 5) able to understand and sign informed consent form in English. Patients with existing or previous stoma will be excluded.

### Study intervention

A self-administered rectal cancer surgery decision aid that was developed according to the International Patient Decision Aid Standards (IPDAS) to be used prior to the consultation with the surgeon. During development, a systematic review on long-term postoperative bowel dysfunction and needs assessment were performed and indicated a need for a rectal cancer surgical decision aid. Its content was informed by the highest evidence from existing literature. The decision tool synthesized information on options and their benefits and harms, a values-clarification exercise and guidance in the steps of decision making. The goal of the decision aid is to enhance patients’ knowledge of surgical options, clarify their values associated with each option, and thus foster decisions that are value-congruent. The decision aid is available in paper-based and web-based interactive formats, using both figures and statistics to convey evidence-based knowledge. Patients will receive both versions of the decision aid as part of the intervention and may choose to use one, or both formats. This decision aid will be used as an adjunct to counseling with the health care team.

On the day of return to clinic for surgical consent, a single page summary sheet will be created based on collected patient data for use by the surgeon and patient.

### Procedure

Recruitment will occur at the initial visit at a Cancer Assessment Centre. After completing consent, patients will answer a pre-decision aid questionnaire on paper for baseline measures on knowledge, preferred choice, and decisional conflict while in clinic. Participants are then asked to view the decision aid (paper-based, web-based, or both) in its entirety (Additional file 1) at home, and answer a post-decision aid questionnaire (Additional file 1) (on paper, on the web, or both) that assesses the acceptability of the decision aid, knowledge, value, decisional conflict, preferred choice, and preparation for decision-making. In this study, we will aim to obtain answers from participants within three weeks of their initial visit at the cancer centre by placing a phone call. This process can limit the time lapse between the viewing of the decision aid and answering the post-PtDA questions. Patients who do not view the PtDA or who are difficult to contact within three weeks, will be encouraged to send in the answers in a stamped envelope included in their package.

### Instruments

The instruments are used to measure both the choices and decision making processes among patients, the acceptability of the decision aid, and feasibility of its implementation into routine clinical practice. Patient choice will be measured by the PtDA effect on their knowledge, values, and preferred choice. Decision making process will be measured by patients’ decisional conflict and preparedness to have a discussion with a healthcare professional. The primary outcome, decisional conflict scale (DCS) [37] -will be measured using the 16 item 5 response category instrument. It is robust in construct validity, reliability with a Cronbach’s alpha coefficient exceeding 0.78, and is sensitive to change when used as a single construct [38]. The scale is also subcategorized into uncertainty, informed, values clarity, support, and effectiveness. The value tool was specifically developed for the decision aid following ODSF guidelines (Additional file 1). It is a 6 item scale measuring importance placed on values associated with bowel anastomosis and permanent stoma. Answering theses value items will help clarify patient values associated with each surgical option. The choice predisposition tool looks at any inclination towards an option before and after administering the PtDA [39]. It is measured by a 15 point scale ranging from −7 (towards stoma) to +7 (towards bowel hookup). It has a test retest coefficient of 0.90 [40] and is sensitive to change in particularly the undecided participants [41-43]. The preparation for decision making tool is a 10 item scale examines a patient’s perception on how the PtDA has prepared them to communicate with practitioner [44]. This tool has a high Cronbach’s alpha coefficient of 0.92 to 0.96 and total reliability of 0.944 [45]. The acceptability tool is measured by a 7 item scale testing the comprehensibility of components of a PtDA, including its length, pace, amount of information, balance in presentation, and overall suitability [46]. This tool has face validity and has been used in at least 8 studies [46]. The decisional conflict, choice predisposition, preparation for decision making scales, and acceptability tools were obtained from the Ottawa Decision Support Group (ODSF) [47].

### Sample size

A convenience sample of patients at The Ottawa Hospital who have rectal cancer and meet the eligibility criteria will be invited to participate. A paired t-test will be used to compare the means of ‘decisional conflict’ score measured before and after the PtDA implementation. Assuming a significance level of 0.05, power of 0.80, an expected standard deviation of 0.6 [38], our required sample size is 34 to detect a clinically relevant difference of 0.3 in the decisional conflict score. The minimal clinical difference for the DCS scale was previously established because this difference is able to discriminate people who make decisions from those who delay decisions in a validation study by O’Connor [37]. Accounting for a drop out rate of 10%, our estimated sample size is 38. The study will continue until the recruitment target of 38 patients has been reached.

### Outcomes

#### Primary outcome measure

The primary outcome will be decisional conflict and knowledge of surgical options. The decisional conflict scale and knowledge test will be used before and after decision aid. The mean scores will be compared using paired t-test. Knowledge test will be a score on 4 questions reflecting key points in knowledge to make decisions.

#### Secondary outcome measures

Secondary outcomes will be measured on choice predisposition, value, preparation for decision making of the patients, acceptability of the tool, and feasibility of DA delivery as part of usual clinical practice.

Acceptability of the decision aid among patients, value score related to each surgical option, and preparation for decision making score will be summarized and described. Preferred choice will be measured before and after the DA administration and the proportion of people with change in preference will be described. Feasibility of DA delivery will be described as manifest through mean time spent on the pre-decision aid questionnaire and the impact on subsequent length of surgical consent visit.

Data will be entered and stored in a database on a password-protected computer on site. Data checking will be performed to identify duplications and missing data. Strategies including case-wise deletion, variable deletion, and imputation will be considered to handle missing data. Analysis will be done through SAS 9.2 statistical software.

### Ethics approval

This project has been approved by the Ottawa Health Science Network Research Ethics Board.

### Part 2: survey of surgeons and nursing staff

Research question: What are the perceived facilitators and barriers influencing the implementation of the rectal cancer decision aid according to the healthcare providers?

### Study design

A web-based survey will be guided by the Knowledge to Action Framework [33] using the survey implementation method proposed by Couper on Internet survey design [48]. Knowledge to Action Cycle [34] states that barriers to knowledge use are important to determine prior to implementation of knowledge translation tools such as patient decision aids in clinical practice [33]. An internet survey is the most feasible option to reach the broad target population of all Canadian colorectal surgeons working with rectal cancer patients. Consistent with other survey methodologists, Couper suggests that survey implementation should include three stages in pre-notification, invitation to survey, and follow-up. The current study will include all three stages.

### Sampling frame

Colorectal surgeons in Canada can be identified by membership with the Canadian Society of Colon and Rectal Surgeons (CSCRS). This organization has been contacted and a list of members’ updated emails are maintained and accessible. There are an estimated 114 surgeons registered with CSCRS. Eligible surgeons working specifically with rectal cancer patients will be identified by the first question in the online survey.

### Procedure

An email will be sent to all surgeons in the directory. Publicly available member lists will be compared to eliminate potential duplicates. An online survey will be created via Fluid Surveys. To maximize the response rate, the survey implementation will follow recommendations of Couper [48], consisting of a pre-notification email, a personalized email invitation to participate with the decision aid attached and a link to the online survey, a reminder email will be sent on days three and six following the invitation email. A final email reminder to non-responders will be sent to further encourage response on day ten.

### Instrument

A single survey tool modified from the Facilitators and Barriers Survey by Graham et al. will be used for physicians [49]. The 41 item survey will be divided into five main areas: 1) development of the decision aid; 2) content and format of the decision aid; 3) decision aid and meeting patients’ needs; 4) physicians’ clinical practice; 5) implementation. All questions will be rated on a 5-point scale from 1 (strongly agree) to 5 (strongly disagree). There are two questions asking surgeons how likely they are to use the decision aid, rated as “not at all” to “very likely”. Two questions will explore the additional facilitators and barriers not mentioned in the survey. Two questions will explore program specific adaptation and further comments. This survey tool was first used in a study of 270 physicians in three different specialties and later modified for two subsequent studies of nurses. Using principal component analysis with Verimax rotation, Graham et al. showed factorial validity with survey items loaded on four components including quality and value for patients, value for physicians, decision aid content, and implementation issues [49]. The same survey tool was then adapted and used in studies to assess factors influencing nurses’ use of decision aid in a primary care call center [50] and an Australian cancer call center [51].

### Outcomes

The response rate will be determined. Common barriers and facilitators to the implementation of the decision aid will be identified. The primary outcome is intention to implement the decision aid. The response distribution will be analysed, and we will identify factors affecting the intention to implement the decision aid.

### Statistical analysis

Data will be captured by the online survey software and downloaded to the study database. Response rate will be determined based on the total number of completed, partially completed, refusals and breakoffs, noncontact (failed to deliver), and unknown eligibility. Ineligibility will be determined by the first question on the survey asking respondents to identify whether they are surgeons working with rectal cancer patients. Non-response rate will be reported. Descriptive statistics will be used to generate characteristics of survey respondents. All response distribution will be presented.

The intention question on the physicians’ likelihood of using the decision aid will be dichotomized into a binary variable with ‘likely’ and ‘very likely’ versus the rest of the responses. Intention will be subsequently used as a response variable in a logistic regression model. Principal component analysis with Verimax rotation will be performed on all items to confirm common themes and logistic regression analysis will be used to identify independent variables associated with the intention to use the decision aid. The independent variables will be components extracted from principal component analysis.

Item-missing data will be explored and addressed by casewise deletion, variable deletion, and possibly imputation, depending on its extent and nature. Respondents presenting with more than half missing values will be excluded from analysis.

## Discussion

Shared decision making is a strategy that is being promoted across the healthcare systems in the US and Canada [52]. Currently the rectal cancer decision aid has been developed, but has not been formally evaluated. This study aims to evaluate the decision aid and explore the perceived barriers and facilitators to the implementation of the decision aid. It will help with planning strategies for designing interventions to facilitate implementation and overcome known barriers to use of the decision aid in routine clinical practice [32].

Physician/nursing/management collaboration will also be a major factor in completing the pre- and post- study. We will hold meetings with surgeons and nursing staff at the Cancer Assessment Centre to increase awareness among staff. Information sheets will be available in the rectal cancer clinics to remind the staff of inclusion & exclusion criteria of the study.

The single arm pre- and post- design of the study and the lack of control and blinding pose threat to our ability to make causal inferences. Nonetheless, important information will be obtained on how the rectal cancer DA may impact the choice and decision-making process of a patient, the acceptability of the decision tool to patients, and the feasibility of integrating this decision aid into everyday practice.

For the survey study, low survey response rate may introduce non-response bias where responders and non-responders are different. Therefore, this study will attempt to maximize response rate by obtaining accurate email addresses, using a simple and concise questionnaire, and sending follow up reminders [48]. Response/non-response will be reported. Discussion of possible bias will be an important part of the interpretation of the findings.

The implementation of the decision aid could lead to more evidence based knowledge dissemination related to rectal cancer and the surgical choices, less uncertainty related to decisions, and ultimately decisions more congruent with patients’ values. To our knowledge this is the first study designed to evaluate a decision aid in the field of colorectal oncology. The data collected from this study could lead to more definitive large scale studies on the surgical decision aid in the future.

## Competing interest

The authors declare that they have no financial or non-financial competing interests.

## Authors’ contributions

RW was involved in the design of the study, drafting the manuscript, and will be accountable for the accuracy and integrity of the research. RB contributed to the design and content of the decision aid and is involved in design and conduct of the study. RB critically reviewed the manuscript. BP contributed to the design and methodology of the study. BP critically reviewed the manuscript. DS was involved in the design and content of the decision aid, the methodology of the study. DS critically reviewed the manuscript. All authors read and approved the final manuscript.

## Pre-publication history

The pre-publication history for this paper can be accessed here:

http://www.biomedcentral.com/1471-2482/14/16/prepub

## Supplementary Material

Additional file 1 The Rectal Cancer Decision Aid and Post-Decision Aid Questionnaire. Click here for file

## References

[B1] Canadian Cancer Society’s Advisory Committee on Cancer Statistics Canadian Cancer Statistic 2012 2012 Toronto

[B2] Perry WB Connaughton JC Abdominoperineal resection: how is it done and what are the results? Clin Colon Rectal Surg 2007 1 213 220 20011202 10.1055/s-2007-984865 PMC2789508

[B3] Allal AS Gervaz P Gertsch P Bernier J Roth AD Morel P Bieri S Assessment of quality of life in patients with rectal cancer treated by preoperative radiotherapy: a longitudinal prospective study Int J Radiat Oncol Biol Phys 2005 61 1129 1135 10.1016/j.ijrobp.2004.07.726 15752893

[B4] de Campos-Lobato LF Alves-Ferreira PC Lavery IC Kiran RP Abdominoperineal resection does not decrease quality of life in patients with low rectal cancer Clinics (Sao Paulo) 2011 66 1035 1040 10.1590/S1807-59322011000600019 21808871 PMC3129949

[B5] Celasin H Karakoyun R Yılmaz S Elhan AH Erkek B Kuzu MA Quality of life measures in Islamic rectal carcinoma patients receiving counselling Colorectal Dis 2011 13 e170 e175 10.1111/j.1463-1318.2011.02649.x 21651692

[B6] Varpe P Huhtinen H Rantala A Salminen P Rautava P Hurme S Grönroos J Quality of life after surgery for rectal cancer with special reference to pelvic floor dysfunction Colorectal Dis 2011 13 399 405 10.1111/j.1463-1318.2009.02165.x 20041930

[B7] Cornish J Tilney HS Heriot AG Lavery IC Fazio VW Tekkis PP A meta-analysis of quality of life for abdominoperineal excision of rectum versus anterior resection for rectal cancer Ann Surg Oncol 2007 14 2056 2068 10.1245/s10434-007-9402-z 17431723

[B8] Pachler J Quality of life after rectal resection for cancer, with or without permanent colostomy. Cochrane Database Syst Rev 2012 CD004323 15266529 10.1002/14651858.CD004323.pub2

[B9] Howie JG Heaney DJ Maxwell M Walker JJ Freeman GK Developing a “consultation quality index” (CQI) for use in general practice Fam Pract 2000 17 455 461 10.1093/fampra/17.6.455 11120715

[B10] Barry MJ Edgman-Levitan S Shared decision making–pinnacle of patient-centered care N Engl J Med 2012 366 780 781 10.1056/NEJMp1109283 22375967

[B11] Salzburg Global Seminar Salzburg statement on shared decision making BMJ 2011 342 December 2010 d1745 21427038 10.1136/bmj.d1745

[B12] National Academy for State Health Policy Shared Decision Making: Advancing Patient-Centered Care through State and Federal Implementation 2012 Washington, DC

[B13] O’Connor AM Bennett C Stacey D Barry MJ Col NF Eden KB Entwistle V Fiset V Holmes-Rovner M Khangura S Llewellyn-Thomas H Rovner DR Do patient decision aids meet effectiveness criteria of the international patient decision aid standards collaboration? A systematic review and meta-analysis Med Decis Making 2007 27 554 574 10.1177/0272989X07307319 17873255

[B14] Stacey D Bennett CL Barry MJ Col NF Eden KB Holmes-Rovner M Llewellyn-Thomas H Lyddiatt A Légaré F Thomson R Decision aids for people facing health treatment or screening decisions Cochrane Database Syst Rev 2011 CD001431 21975733 10.1002/14651858.CD001431.pub3

[B15] O’Brien MA Whelan TJ Villasis-Keever M Gafni A Charles C Roberts R Schiff S Cai W Are cancer-related decision aids effective? A systematic review and meta-analysis J Clin Oncol 2009 27 974 985 10.1200/JCO.2007.16.0101 19124808

[B16] Patient decision aids [http://decisionaid.ohri.ca/index.html]

[B17] Elwyn G O’Connor A Stacey D Volk R Edwards A Coulter A Thomson R Barratt A Barry M Bernstein S Butow P Clarke A Entwistle V Feldman-Stewart D Holmes-Rovner M Llewellyn-Thomas H Moumjid N Mulley A Ruland C Sepucha K Sykes A Whelan T Developing a quality criteria framework for patient decision aids: online international Delphi consensus process BMJ 2006 333 417 10.1136/bmj.38926.629329.AE 16908462 PMC1553508

[B18] O’Connor a M Tugwell P Wells G a Elmslie T Jolly E Hollingworth G McPherson R Bunn H Graham I Drake E A decision aid for women considering hormone therapy after menopause: decision support framework and evaluation Patient Educ Couns 1998 33 267 279 10.1016/S0738-3991(98)00026-3 9731164

[B19] Connor AMO Jacobsen MJ Stacey D An Evidence-Based Approach to Managing Women’s Decisional Conflict J Obstet Gynecol Neonatal Nurs 2002 31 570 581 10.1111/j.1552-6909.2002.tb00083.x 12353737

[B20] Connor AO Jacobsen MJ Workbook on developing and evaluating patient decision aids 2003 [https://decisionaid.ohri.ca/docs/develop/Develop_DA.pdf]

[B21] Brinkman B Lawson M Patient Decision Aids Based on ODSF: A Synthesis of Findings from 24 RCT 2010 Ottawa [https://decisionaid.ohri.ca/docs/ODSF-workshop/ODSF-PatientDecisionAids-Lawson-Brinkman.pdf]

[B22] Scheer AS Boushey RP Liang S Doucette S O’Connor AM Moher D The long-term gastrointestinal functional outcomes following curative anterior resection in adults with rectal cancer: a systematic review and meta-analysis Dis Colon Rectum 2011 54 1589 1597 10.1097/DCR.0b013e3182214f11 22067190

[B23] Scheer AS O’Connor AM Chan BPK Moloo H Poulin EC Mamazza J Auer RC Boushey RP The myth of informed consent in rectal cancer surgery: what do patients retain? Dis Colon Rectum 2012 55 970 975 10.1097/DCR.0b013e31825f2479 22874604

[B24] Légaré F Ratté S Gravel K Graham ID Barriers and facilitators to implementing shared decision-making in clinical practice: update of a systematic review of health professionals’ perceptions Patient Educ Couns 2008 73 526 535 10.1016/j.pec.2008.07.018 18752915

[B25] Légaré F Turcotte S Stacey D Patients’ perceptions of sharing in decisions Patient Patient- … 2012 5 1 19 10.2165/11592180-000000000-00000 22276987

[B26] Nannenga MR Montori VM Weymiller AJ Smith SA Christianson TJH Bryant SC Gafni A Charles C Mullan RJ Jones LA Bolona ER Guyatt GH A treatment decision aid may increase patient trust in the diabetes specialist. The Statin choice randomized trial Health Expect 2009 12 38 44 10.1111/j.1369-7625.2008.00521.x 19250151 PMC5060475

[B27] Mullan RJ Montori VM Shah ND Christianson TJH Bryant SC Guyatt GH Perestelo-Perez LI Stroebel RJ Yawn BP Yapuncich V Breslin MA Pencille L Smith SA The diabetes mellitus medication choice decision aid: a randomized trial Arch Intern Med 2009 169 1560 1568 19786674 10.1001/archinternmed.2009.293

[B28] Holmes-Rovner M Valade D Orlowski C Draus C Nabozny-Valerio B Keiser S Implementing shared decision-making in routine practice: barriers and opportunities Health Expect 2000 3 182 191 10.1046/j.1369-6513.2000.00093.x 11281928 PMC5080967

[B29] O’Connor AM Graham ID Visser A Implementing shared decision making in diverse health care systems: the role of patient decision aids Patient Educ Couns 2005 57 247 249 10.1016/j.pec.2005.04.010 15893205

[B30] Towle A Godolphin W Grams G Lamarre A Putting informed and shared decision making into practice Health Expect 2006 9 321 332 10.1111/j.1369-7625.2006.00404.x 17083559 PMC5060372

[B31] O’Donnell S Cranney A Jacobsen MJ Graham ID O’Connor AM Tugwell P Understanding and overcoming the barriers of implementing patient decision aids in clinical practice J Eval Clin Pract 2006 12 174 181 10.1111/j.1365-2753.2006.00613.x 16579826

[B32] Straus S Knowledge Translation in Healthcare: Moving from Evidence to Practice 2009 West Sussex, England: Blackwell Publishing Ltd

[B33] Graham ID Logan J Harrison MB Straus SE Tetroe J Caswell W Robinson N Lost in knowledge translation: time for a map? J Contin Educ Health Prof 2006 26 13 24 10.1002/chp.47 16557505

[B34] KT Clearinghouse http://ktclearinghouse.ca/knowledgebase/knowledgetoaction

[B35] Solberg LI Guideline implementation: why don’t we do it? Am Fam Physician 2002 65 176 181–2 11820484

[B36] O’Connor AM Fiset V DeGrasse C Graham ID Evans W Stacey D Laupacis A Tugwell P Decision aids for patients considering options affecting cancer outcomes: evidence of efficacy and policy implications J Natl Cancer Inst Monogr 1999 9 67 80 10854460 10.1093/oxfordjournals.jncimonographs.a024212

[B37] O’Connor AM Validation of a decisional conflict scale Med Decis Making 15 25 30 7898294 10.1177/0272989X9501500105

[B38] O’Connor AM User Manual – Decisional Conflict Scale (16 Item Question Format) 1993 [https://decisionaid.ohri.ca/docs/develop/User_Manuals/UM_Decisional_Conflict.pdf]

[B39] O’Connor A User manual-measures of decision/choice predisposition 2003 [http://decisionaid.ohri.ca/docs/develop/User_Manuals/UM_ChoicePredisposition_Decision.pdf]

[B40] O’Connor AM Tugwell P Wells GA Elmslie T Jolly E Hollingworth G McPherson R Drake E Hopman W Mackenzie T Randomized trial of a portable, self-administered decision aid for postmenopausal women considering long-term preventive hormone therapy Med Decis Making 1998 18 295 303 10.1177/0272989X9801800307 9679994

[B41] Cranney A O’Connor AM Jacobsen MJ Tugwell P Adachi JD Ooi DS Waldegger L Goldstein R Wells GA Development and pilot testing of a decision aid for postmenopausal women with osteoporosis Patient Educ Couns 2002 47 245 255 10.1016/S0738-3991(01)00218-X 12088603

[B42] Stacey D O’Connor AM DeGrasse C Verma S Development and evaluation of a breast cancer prevention decision aid for higher-risk women Health Expect 2003 6 3 18 10.1046/j.1369-6513.2003.00195.x 12603624 PMC5060163

[B43] Mitchell SL Tetroe J O’Connor AM A decision aid for long-term tube feeding in cognitively impaired older persons J Am Geriatr Soc 2001 49 313 316 10.1046/j.1532-5415.2001.4930313.x 11300244

[B44] Graham ID O’Connor A User Manual-Preparation for Decision Making Scale 2010 10.1016/j.pec.2009.05.012 19560303

[B45] Bennett C Graham ID Kristjansson E Kearing SA Clay KF O’Connor AM Validation of a preparation for decision making scale Patient Educ Couns 2010 78 130 133 10.1016/j.pec.2009.05.012 19560303

[B46] O’Connor A Cranney A User Manual – Acceptability. 1996 2002 1 5

[B47] Ottawa decision support framework http://decisionaid.ohri.ca/eval.html

[B48] Couper MP Designing Effective Web Surveys 2008 New York: Cambridge University Press

[B49] Graham ID Logan J Bennett CL Presseau J O’Connor AM Mitchell SL Tetroe JM Cranney A Hebert P Aaron SD Physicians’ intentions and use of three patient decision aids BMC Med Inform Decis Mak 2007 7 20 10.1186/1472-6947-7-20 17617908 PMC1931587

[B50] Stacey D Graham ID O’Connor AM Pomey M-P Barriers and facilitators influencing call center nurses’ decision support for callers facing values-sensitive decisions: a mixed methods study Worldviews Evid Based Nurs 2005 2 184 195 10.1111/j.1741-6787.2005.00035.x 17040526

[B51] Stacey D Chambers SK Jacobsen MJ Dunn J Overcoming barriers to cancer-helpline professionals providing decision support for callers: an implementation study Oncol Nurs Forum 2008 35 961 969 10.1188/08.ONF.961-969 18980928

[B52] Stiggelbout AM Weijden TVD Wit MPTD Frosch D Legare F Montori VM Trevena L Elwyn G Shared decision making: really putting patients at the centre of healthcare BMJ 2012 344 e256 e256 10.1136/bmj.e256 22286508

