# Supplementary material for: The evaluation of a rectal cancer decision aid and the factors influencing its implementation in clinical practice
Source: BMC Surg. 2014 Mar 21;14:16. doi: 10.1186/1471-2482-14-16 (PMC3998110; doi:10.1186/1471-2482-14-16)
Supplement: Additional file 1 — The Rectal Cancer Decision Aid and Post-Decision Aid Questionnaire. [file 1471-2482-14-16-S1.pdf]

Name: ______________________________

Should I have my bowels "hooked up" (anastomosis) when removing my rectal cancer?

A decision aid for **patients with rectal cancer**

This decision aid is for you if:

- You have rectal cancer treatable by surgery
- Your surgeon has suggested having your bowels hooked back up at surgery

What is rectal cancer?

- Rectal cancer is a tumour located in the last 15 cm of the intestine.
- It is a difficult cancer to treat as it grows close to the muscles involved in the control of bowel movements and gas.
- The first goal of treatment is to remove all of the tumour for the best chance of survival.
- The second goal of treatment is to achieve acceptable bowel function.
- Although having your bowels hooked up is commonly recommended there is another option of having a permanent stoma (bag). These options are described next.

What are your options to remove the tumor?

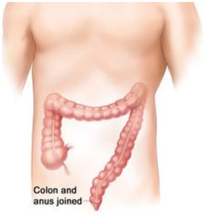

Bowel hook up (Anterior Resection): The rectum is removed and the two healthy ends of bowel are reconnected. Your surgeon may need to create a temporary stoma (bag) to allow the hook up to heal. This temporary stoma produces more liquid stool with little odour. After 4-6 months the temporary stoma is reversed. After recovery, bowel movements are likely to be less predictable, more frequent, including at night, and urgent. Most people are able to manage this change. Some patients have poor control and have another operation for a permanent stoma. Patients who have radiation are more likely to experience changes in their bowel habits.

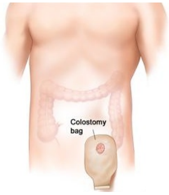

Permanent stoma (Abdominoperineal Resection): The rectum and anus are removed. The area of the anus is permanently closed and the bowel is not hooked up. The bowel is brought out to the skin and a stoma bag is attached. Following recovery, bowel movements will become more regular and predictable (e.g. morning and night). The bag controls smells and release of gas. Daily stoma care is necessary such as cleaning the area, changing the bag and applying creams. The costs of these materials may be covered. Most people resume their normal activities.

Name: ________________________________

What other health factors may affect your choice? Check ☑ any that apply.

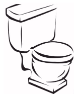

Do you currently have bowel problems?

		Never	Once a week	More than once a week	Daily
1.1	Accidental leaks of gas?	☐	☐	☐	☐
1.2	Accidental leaks of liquid stool?	☐	☐	☐	☐
1.3	Accidental leaks of solid stool?	☐	☐	☐	☐
1.4	Wear a pad because of accidental leaks of liquid or solid stool?	☐	☐	☐	☐

☐ None of these apply to me

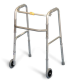

Do you have problems getting to the bathroom?

		Yes	No
1.5	Difficulty walking?	☐	☐
1.6	Use a wheelchair?	☐	☐
1.7	Job/lifestyle limits easy access to bathrooms? (eg. Outdoors)	☐	☐

☐ None of these apply to me

Name: ______________________________

Step 1: What are the benefits and harms of each option?

What does the research show?

Blocks of 100 faces show a ‘best estimate’ of what happens to **100 people** after surgery and up to 4 years after who have their bowels reconnected or a permanent colostomy. Each face(😐) stands for one person. The shaded areas show the number of people affected. There is no way of knowing in advance if you will be one of those affected. You should discuss with your surgeon if you have any risk factors for these outcomes.

	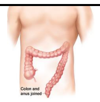		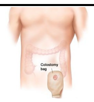	
Benefits	Bowel hook up		Permanent stoma	
85 fewer people have a permanent stoma if they have their bowels reconnected.<br><br>By avoiding a permanent stoma you also avoid the complications of a permanent stoma such as:<br>• hernia of the stoma<br>• bowel sliding out through the stoma<br>• skin irritation around the stoma<br>• problems with healing or infection where the anus was closed	12	get a permanent stoma	100	get a permanent stoma
	85	avoid a permanent stoma	0	avoids a permanent stoma
	😐😐😐😐😐😐😐😐😐😐<br>😐😐😐😐😐😐😐😐😐😐<br>😐😐😐😐😐😐😐😐😐😐<br>😐😐😐😐😐😐😐😐😐😐<br>😐😐😐😐😐😐😐😐😐😐<br>😐😐😐😐😐😐😐😐😐😐<br>😐😐😐😐😐😐😐😐😐😐<br>😐😐😐😐😐😐😐😐😐😐<br>😐😐😐😐😐😐😐😐😐😐<br>😐😐😐😐😐😐😐😐😐😐		😐😐😐😐😐😐😐😐😐😐<br>😐😐😐😐😐😐😐😐😐😐<br>😐😐😐😐😐😐😐😐😐😐<br>😐😐😐😐😐😐😐😐😐😐<br>😐😐😐😐😐😐😐😐😐😐<br>😐😐😐😐😐😐😐😐😐😐<br>😐😐😐😐😐😐😐😐😐😐<br>😐😐😐😐😐😐😐😐😐😐<br>😐😐😐😐😐😐😐😐😐😐<br>😐😐😐😐😐😐😐😐😐😐	
Risks				
3 more people who have their bowels reconnected with a temporary stoma return to the operating room (OR) because of a life-threatening leak at the connection site. This may mean extra treatments, longer hospital stay and a longer recovery. You may need to be treated in the intensive care unit.<br><br>This number is higher for people who do not have a temporary stoma when their bowels are reconnected. In this case, 11 more people will return to the OR.	3	return to the OR	0	return to the OR
	97	avoid this	100	avoid this
	😐😐😐😐😐😐😐😐😐😐<br>😐😐😐😐😐😐😐😐😐😐<br>😐😐😐😐😐😐😐😐😐😐<br>😐😐😐😐😐😐😐😐😐😐<br>😐😐😐😐😐😐😐😐😐😐<br>😐😐😐😐😐😐😐😐😐😐<br>😐😐😐😐😐😐😐😐😐😐<br>😐😐😐😐😐😐😐😐😐😐<br>😐😐😐😐😐😐😐😐😐😐<br>😐😐😐😐😐😐😐😐😐😐		😐😐😐😐😐😐😐😐😐😐<br>😐😐😐😐😐😐😐😐😐😐<br>😐😐😐😐😐😐😐😐😐😐<br>😐😐😐😐😐😐😐😐😐😐<br>😐😐😐😐😐😐😐😐😐😐<br>😐😐😐😐😐😐😐😐😐😐<br>😐😐😐😐😐😐😐😐😐😐<br>😐😐😐😐😐😐😐😐😐😐<br>😐😐😐😐😐😐😐😐😐😐<br>😐😐😐😐😐😐😐😐😐😐	

Name: ______________________________

	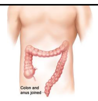	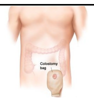
Risks continued	Bowel hook up	Permanent stoma
35 more people will complain of some form of incontinence following bowel hook up. The majority has minor incontinence (accidents) to gas and liquid stool. 14 people (out of 100) experience major incontinence to solid stool.<br><br>All of these people choose to wear a protective pad in their underwear.	35 wear a pad<br>65 avoid a pad<br>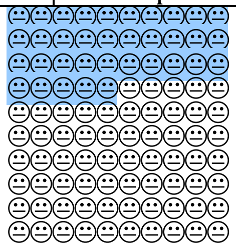	0 wear a pad<br>100 avoid a pad<br>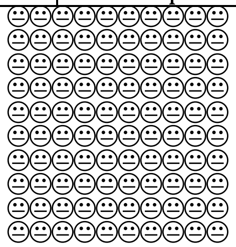
Problems from either surgery:		
Some people who have either surgery report worsening of their urinary or sexual function. The number of people who report this problem is the same for both surgeries.<br><br>Urinary problems include difficulty emptying the bladder. There may also be problems of leaking urine, which is more common in women.<br><br>For men, sexual problems may include impotence or ejaculation problems. For women, sexual problems may include vaginal dryness, pain during intercourse, problems with arousal or achieving orgasm.		

Name: ______________________________

Step 2. Which reasons to choose each option matter most to you?

Common reasons to choose each option are listed below.
Mark ✓ how much each reason matter to you on a scale from 0 to 5.
‘0’ means it is not important to you. ‘5’ means it is very important to you.

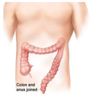

Reasons to…

Choose to have your bowels hooked up

		Not Important		Somewhat Important		Very Important	
		[0]	[1]	[2]	[3]	[4]	[5]
2.1	How important is it for you to have bowel movements the usual way?	☐	☐	☐	☐	☐	☐
2.2	How important is it to you to avoid the inconvenience of caring for a stoma? (e.g. emptying the bag, cleaning the area, applying creams and buying bags)	☐	☐	☐	☐	☐	☐
2.3	How important is it for you to avoid the complications of a permanent stoma? (including wound healing problems, stoma hernias, and skin irritations)	☐	☐	☐	☐	☐	☐

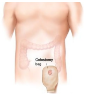

Reasons to ...

Choose a permanent stoma

		Not Important		Somewhat Important		Very Important	
		[0]	[1]	[2]	[3]	[4]	[5]
2.4	How important is it that your bowel movements are more predictable, less frequent and less urgent?	☐	☐	☐	☐	☐	☐
2.5	How important is it for you to avoid incontinence (leaking) of stool or gas?	☐	☐	☐	☐	☐	☐
2.6	How important is it to for you to avoid having multiple surgeries?	☐	☐	☐	☐	☐	☐

Name: ______________________________

List other reasons that are important	☐	☐	☐	☐	☐	☐
List other reasons that are important	☐	☐	☐	☐	☐	☐

Now, thinking about the reasons that are most important to you...

3.1 If you definitely prefer a permanent stoma, check ☑ the box far to the right.
If you definitely prefer a bowel hook up, check ☑ the box far to the left.
If you are uncertain, check ☑ the centre box

☐	☐	☐	☐	☐	☐	☐	☐	☐	☐	☐	☐	☐	☐	☐
Bowel hook up							Uncertain							Permanent stoma

Choice predisposition © AM O'Connor 1996

Step 3: What else do you need to prepare for decision-making?

KNOWLEDGE

Find out how well this decision aid helped you learn the key facts.

	Check ☑ the best answer.	**Bowel hook up**	**Permanent stoma**	**Don't know**
4.1.	Which option has the highest chance of needing a second surgery?	☐	☐	☐
4.2	Which option cannot be reversed?	☐	☐	☐
4.3	Which option has the highest chance of hernia and wound healing problems?	☐	☐	☐
4.4	Which option has the highest chance of incontinence (accidents) of stool or gas?	☐	☐	☐

		**Yes**	**No**
4.5	Do you know enough about the benefits and side effects of each option?	☐	☐

Check your answers at the bottom of the page

Name: ______________________________

Decisional Conflict

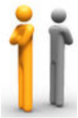

Find out if you have uncertainties.

		Strongly agree [0]	Agree [1]	Neither Agree Nor Disagree [2]	Disagree [3]	Strongly Disagree [4]
5.1	I know which options are available to me.	☐	☐	☐	☐	☐
5.2	I know the benefits of each option.	☐	☐	☐	☐	☐
5.3	I know the risks and side effects of each option.	☐	☐	☐	☐	☐
5.4	I am clear about which benefits matter most to me.	☐	☐	☐	☐	☐
5.5	I am clear about which risks and side effects matter most to me.	☐	☐	☐	☐	☐
5.6	I am clear about which is more important to me (the benefits or the risks and side effects).	☐	☐	☐	☐	☐
5.7	I have enough support from others to make a choice.	☐	☐	☐	☐	☐
5.8	I am choosing without pressure from others.	☐	☐	☐	☐	☐
5.9	I have enough advice to make a choice.	☐	☐	☐	☐	☐
5.10	I am clear about the best choice for me.	☐	☐	☐	☐	☐
5.11	I feel sure about what to choose.	☐	☐	☐	☐	☐
5.12	This decision is easy for me to make.	☐	☐	☐	☐	☐

Name: ______________________________

		Strongly agree	Agree	Neither Agree Nor Disagree	Disagree	Strongly Disagree
		[0]	[1]	[2]	[3]	[4]
5.13	I feel I have made an informed choice.	☐	☐	☐	☐	☐
5.14	My decision shows what is important to me.	☐	☐	☐	☐	☐
5.15	I expect to stick with my decision.	☐	☐	☐	☐	☐
5.16	I am satisfied with my decision.	☐	☐	☐	☐	☐

Decisional conflict scale © AM O'Connor 2005

The information is not intended to replace the advice of a healthcare provider.
A final decision can only be achieved through discussion with a healthcare provider.

Step 4. What are the next steps?

PREPARATION FOR DECISION MAKING

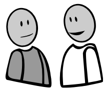

Find out if you are ready to communicate with the doctor.

	**Did this educational material . . .**	Not at all	A little	Some-what	Quite a bit	A great deal
6.1	Help you recognize that a decision needs to be made	☐	☐	☐	☐	☐
6.2	Help you think about the pros and cons of each option?	☐	☐	☐	☐	☐
6.3	Help you think about which pros and cons are most important?	☐	☐	☐	☐	☐
6.4	Help you organize your own thoughts about the choices?	☐	☐	☐	☐	☐
6.5	Help you think about how involved you want to be in this decision?	☐	☐	☐	☐	☐
6.6	Help you identify questions you want to ask your doctor?	☐	☐	☐	☐	☐
6.7	Prepare you to talk to your doctor about what matters most to you?	☐	☐	☐	☐	☐
6.8	Prepare you for a follow-up visit with your doctor?	☐	☐	☐	☐	☐

Answers for the key facts: 1. Bowel hook up 2. Permanent stoma 3. Permanent stoma 4. Bowel reconnection

Content editors: AS Scheer, RC Wu, RP Boushey, PL Roberts
Funded in part by: PSI, ASCRS. All Authors have declared no conflict of interest.

Format based on the Ottawa Personal Decision Guide © 2000, A O'Connor, D Stacey, University of Ottawa, Canada.

Publication date: [date published]. For more information contact the corresponding author: AS Scheer, ascheer@toh.on.ca. Technical information available at url (tbd).

Readability: FK Grade level 8.

References:

1. Matthiessen P, Hallbook O, Rutegard J, Simert G, Sjodahl R. Defunctioning stoma reduces symptomatic anastomotic leakage after low anterior resection of the rectum for cancer: A randomized multicenter trial. Ann Surg 2007: 246: 207 – 214.

2. Carne PWG, Robertson GM, Frizelle FA. Parastomal hernia. British Journal of Surgery 2003; 90: 784-793.

3. Salvadalena G. Incidence of complications of the stoma and peristomal skin among individuals with colostomy, ileostomy, and urostomy: A systematic review. Journal of wound, ostomy, and continence 2008; 35 (6): 596-607.

4. Tan WS, Tang CL, Shi L, Eu KW. Meta-analysis of defunctioning stomas in low anterior resection for rectal cancer. British J of Surg 2009; 96(5): 462 – 472.

5. Scheer AS, Boushey RP, Liang S, Doucette S, O'Connor AM, Moher D. The long-term gastrointestinal functional outcomes following anterior resection for rectal cancer: A systematic review, meta-analysis and meta-regression. Submitted for publication.

6. Lange MM, Mass CP, Marijnen CAM, Wiggers T, Rutten HJ, Klein Kranenberg E, van de Velde CJH, cooperative clinical investigators of the Dutch Total Mesorectal Excision Trial. Urinary dysfunction after rectal cancer treatment is mainly caused by surgery. British J Surg 2008; 95: 1020 – 1028.

7. Lange MM et al. Risk factors for sexual dysfunction after rectal cancer treatment. Eur J Cancer 2009; 45(9): 1578 – 88.

Name: ______________________________

Your feedback on the decision aid

We would like to know what you think about the education package you have just received.

1. Please rate each section, by circling 'poor', 'fair', 'good', or 'excellent' to show what you think about the way the information was presented on:

General information	poor fair good Excellent
Surgical options	poor fair good Excellent
Risks and benefits	poor fair good Excellent
Questions and answers	poor fair good Excellent

2. The length of presentation was (*check one)*

|__| too long

|__| too short

|__| just right

3. The amount of information was (*check one*)

|__| too much information

|__| too little information

|__| just right

4. I found the presentation (*check one*)

|__| slanted towards taking permanent stoma

|__| slanted towards taking bowel hookup

|__| balanced

5. How clear is the information in the decision aid? Check ☑ the best answer

|__| Everything was clear

|__| Most things were clear

|__| Completely balanced

|__| Some things were unclear

Name: ______________________________

|__| Many things were unclear

6. Would you recommend this decision aid to patients? Check ☑ the best answer

|__| I would definitely recommend it

|__| I would probably recommend it

|__| I would probably not recommend it

|__| I would definitely not recommend it

Comments:

7. What did you <u>like</u> about the decision aid?

8. What <u>suggestions do you have to improve</u> the decision aid?

9. Which version of decision aid did you prefer? (Choose paper version or web version)

What is the highest level of education you have achieved?

|__| High school or less

|__| Trade certificate/diploma

|__| College diploma/degree

|__| University degree

Acceptability scale © AM O'Connor, A Cranney 2000
